# Spontaneous Enhancement of Magnetic Resonance Signals Using a RASER

**DOI:** 10.1002/anie.202108306

**Published:** 2021-08-13

**Authors:** Sergey Korchak, Lukas Kaltschnee, Riza Dervisoglu, Loren Andreas, Christian Griesinger, Stefan Glöggler

**Affiliations:** ^1^ NMR Signal Enhancement Group Max Planck Institute for Biophysical Chemistry Am Fassberg 11 37077 Göttingen Germany; ^2^ Center for Biostructural Imaging of Neurodegeneration of UMG Von-Siebold-Str. 3A 37075 Göttingen Germany; ^3^ Research Group for Solid State NMR Max Planck Institute for Biophysical Chemistry Am Fassberg 11 37077 Göttingen Germany; ^4^ Department of NMR-based Structural Biology Max Planck Institute for Biophysical Chemistry Am Fassberg 11 37077 Göttingen Germany

**Keywords:** hyperpolarization, NMR spectroscopy, signal enhancement, small molecules

## Abstract

Nuclear magnetic resonance is usually drastically limited by its intrinsically low sensitivity: Only a few spins contribute to the overall signal. To overcome this limitation, hyperpolarization methods were developed that increase signals several times beyond the normal/thermally polarized signals. The ideal case would be a universal approach that can signal enhance the complete sample of interest in solution to increase detection sensitivity. Here, we introduce a combination of para‐hydrogen enhanced magnetic resonance with the phenomenon of the RASER: Large signals of para‐hydrogen enhanced molecules interact with the magnetic resonance coil in a way that the signal is spontaneously converted into an in‐phase signal. These molecules directly interact with other compounds via dipolar couplings and enhance their signal. We demonstrate that this is not only possible for solvent molecules but also for an amino acid.

## Graphical abstract

We introduce the combination of para‐hydrogen enhanced magnetic resonance with the phenomenon of the RASER and spontaneous polarization transfer: Large signals of para‐hydrogen enhanced molecules interact with the magnetic resonance coil in a way that the signal is spontaneously converted into an in‐phase signal. These molecules directly interact with other compounds via dipolar couplings and enhance their signal.

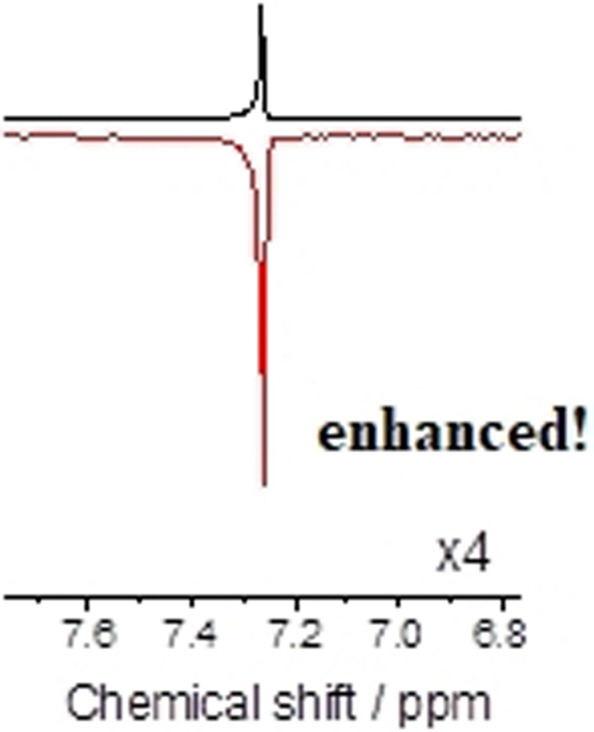

## Introduction

Maximizing the detectable signals is the goal for just about any spectroscopic technique. In particular, the method of nuclear magnetic resonance (NMR) is primarily sensitivity limited.^[1]^ There are several ways to improve sensitivity, such as increasing the magnetic field or cryo‐cooling the detector and generally reducing the noise generated by the spectrometer electronics.^[2]^ Another possibility is to use hyperpolarization methods that increase the spin population in one specific energy state resulting in a signal enhancement as compared with the equilibrium polarization in the NMR magnet.[^2b^, ^3^]

There are several ways to prepare highly polarized nuclear spins. The most universal, solid‐state dynamic nuclear polarization (DNP), can theoretically polarize any kind of molecules in solids but the performance of the method is still strongly dependent on optimization of parameters such as the particular glass matrix forming solvent, molecular structure of the analyte, the choice of polarizing agent, often an organic (bi)radical, and it requires cryogenic working‐temperatures for biological samples.[^3f^, ^4^] An alternative is to enhance signals using para‐hydrogen which can be performed in the liquid state.[^3b^, ^3c^, ^3d^, ^3e^, ^5^] To generate polarization, the singlet spin order of para‐enriched dihydrogen (pH_2_) is used either by reacting pH_2_ with an unsaturated precursor (para‐hydrogen induced polarization, PHIP) or by a reversible exchange process with a mediating catalyst during which the molecule of interest stays unchanged (signal amplification by reversible exchange, SABRE).[^3b^, ^6^] Both para‐hydrogen techniques are, however, very limited with respect to which molecules can be signal‐enhanced because either an unsaturated precursor (PHIP) needs to be available or the molecule of interest must have favorable binding properties to the applied catalyst (SABRE). To further broaden the applicability for both methods, possibilities were devised to signal enhance mobile protons that exchange with another molecule of interest to which the polarization is subsequently transferred.^[7]^ Overall, it would be desirable to obtain a general way of enhancing molecules in the liquid.

## Results and Discussion

Herein, we have used the recently introduced para‐hydrogen RASER (radiofrequency amplification by stimulated emission)^[8]^ and demonstrate that it can be used to spontaneously create large amounts of net magnetization from the non‐equilibrium populations created via PHIP. Hereby stimulated emission, caused by interaction of the sample magnetization with the radiofrequency coil, drives a population inversion for transitions of negative polarization, if these polarizations become large enough. The underlying effect is radiation damping (RD).^[9]^ In our case the anti‐phase patterns typically observed after high‐field PHIP, which do not carry net magnetization, are converted into in‐phase patterns which do carry net magnetization.

The aim of this study is to set a framework for a universal method that can be used to signal‐enhance molecules in solution and we demonstrate this with solvent molecules and an N‐acetylated amino acid as test molecules. Hydrogen atoms are spin‐1/2 nuclei with nearly 100 % natural abundance which we aim to signal enhance to a large extend (>10 %) and transfer the obtained polarization to other molecules of interest in their proximity. One particular strength of this approach is that hydrogen as a source is cost‐efficient and widely available. To do the polarization transfer from signal‐enhanced hydrogen to other molecules, the SPINOE effect^[10]^ will be used which will require an in‐phase magnetization. While heteronuclear approaches, mainly with signal‐enhanced xenon^[11]^ have been studied, we focus here on a homonuclear SPINOE. SPINOE transfer efficiency depends on the dynamics of the two interacting molecules, spin source and the spin receiver. Fast dynamics result in negative spin transfer and very slow dynamics result in positive transfer of the spin polarization. The regime in between is the one where SPINOE may result in signal loss rather than enhancement.^[10b]^ With this in mind, if a large enough hyperpolarization is provided, nearly any dynamics regime can be used for signal enhancement. For systems in which dipole‐dipole cross relaxation is the predominant relaxation mechanism, as assumed for ^1^H‐nuclei herein, at least two modes of motion have to be considered when estimating the efficiency of the SPINOE transfer: Rotational diffusion of the molecules, causing polarization loss due to intramolecular dipole‐dipole relaxation and translational diffusion, causing intermolecular dipole‐dipole relaxation, which is both the driving force for the magnetization transfer during the SPINOE as well as a source for polarization loss. Models for both processes are briefly summarized in chapter 2 of the SI.

Even though the experiments described later will involve more than two spins, quite reasonable estimates of the maximum enhancements that can be expected from the simple two spin‐1/2 model described in chapter 3 of the SI. In our case, a slightly more realistic model including two spin‐1/2 for the polarization source, including intramolecular cross‐relaxation and differential leakage, and one spin in the target molecule yields very similar results (5 % deviation for the solvent case discussed below). We make our estimates, assuming cross‐relaxation driven by translational diffusion in the absence of intermediate complex formation between the spins. If a hyperpolarized molecule such as ethyl acetate‐d_6_ could be prepared with a 100 % spin polarization on ^1^H, notable SPINOE enhancement could be expected for other ^1^H spins with long ^1^H‐*t*_1_, even when working in low viscosity solutions. For an example solution containing 100 mM ethyl acetate and 100 mM CHCl_3_ (^1^H‐*t*_1_≈180 s), a maximum enhancement for CHCl_3_ of *ϵ* ${}_{\mathrm{CHCl}{}_{3},\max}$ ≈−73 at *B*_0_=7 T (*P*=0.16 %) could be expected (see chapter 4 SI). For targets with more typical ^1^H‐*t*_1_ for small organic molecules, the maximum achievable enhancements are smaller, but still sizable. Assuming ^1^H‐*t*_1_ ca. 5 s and diffusion coefficients typically found in for example, methanol we still find *ϵ* ${}_{\mathrm{CHCl}{}_{3},\max}$ ≈−15 at *B*_0_=7 T (*P*=0.034 %). For details on our estimations, see section 4 of the SI. So despite the relatively inefficient polarization transfer through intermolecular NOE by translational diffusion in low viscosity solvents, notable enhancements can still theoretically be achieved with para‐hydrogen enhanced substrates, due to the very high proton polarizations.

**Figure 1:**
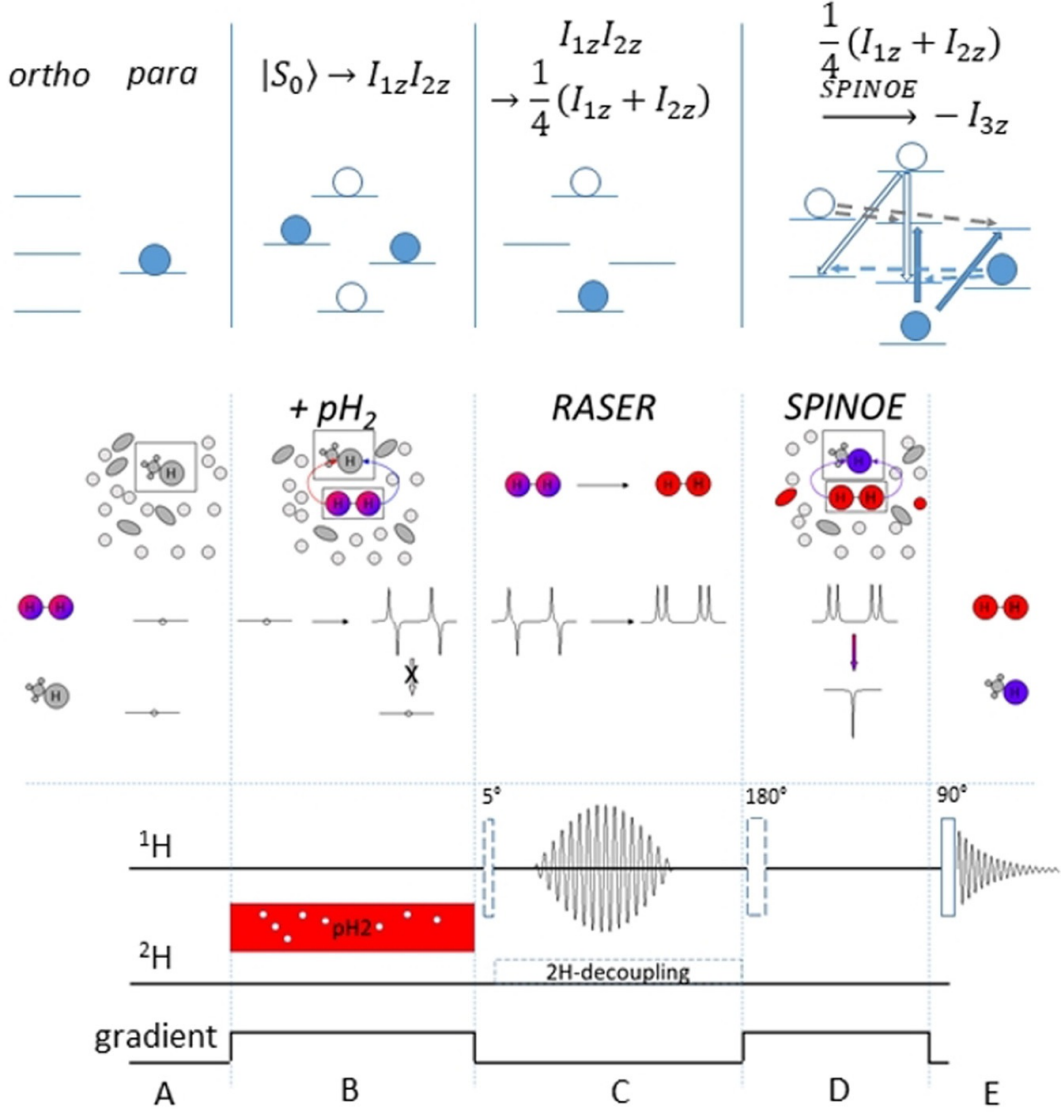
The para‐hydrogen and RASER induced NOE (PRINOE) methodology. Top: representation of molecules and corresponding population. Bottom: synchronized pulse sequence. A) Solution containing an unsaturated moiety in appropriate solvent is fully relaxed inside the spectrometer. B) pH_2_ is added to the thermally polarized sample and a saturated molecule with PASADENA‐type hyperpolarization is created. To prevent the onset of RASER, the reaction is performed in the presence of a static gradient. The created hyperpolarization has an anti‐phase character where hyperpolarized protons, albeit with different NMR chemical shifts, have both absorptive and emissive lines. A SPINOE polarization transfer to other nuclei can result only in zero polarization. C) After the gradient is switched off, the RASER starts spontaneously or can be induced by a 5° pulse. Only negatively polarized lines are affected and evolve making the polarization on both protons purely positive. D) This opens the possibility for intermolecular polarization transfer to nearby nuclei via dipolar interactions resulting in SPINOE‐type hyperpolarization on all other molecules in solution. Optionally a 180° pulse can invert the source of polarization before the SPINOE. E) The resulting polarization pattern is detected by applying a 90° pulse with subsequent acquisition of the free induction decay.

To minimize relaxation losses, the ideal case for enhancing a sample of interest is to perform all the necessary steps directly at the site of measurement which is usually inside a high‐resolution, high‐field spectrometer. The challenge to overcome here is that the resulting proton spin order (after para‐hydrogen is added to an unsaturated bond (PASADENA, parahydrogen and synthesis allow dramatically enhanced nuclear alignment)) results in an anti‐phase term, that is, a mixture of absorptive and emissive signals with a total integral of zero. This behavior is depicted in Figure 1. Each proton has both positive and negative polarization depending on the spin state of the second proton, thus no SPINOE is possible (Figure 1, A and B).

PHIP at low magnetic fields and then transport it into high field (ALTADENA, adiabatic longitudinal transport after dissociation engenders net alignment) results in net polarization on each proton with opposite sign. Hence, when detected in a cryomagnet the sum of both of the observed signals is still zero. We note that theoretically, ALTADENA‐type polarization may produce non‐zero SPINOE when two protons have different intermolecular dipolar couplings to the target spin. Thus, a method to obtain net magnetization of the same sign in large quantities is desired. Using for example, selective pulses is demanding since separation of emissive and absorptive signals is on the order of the small mutual proton *J*‐couplings. Broadband pulse sequences like PHIP‐echo or others can indeed deliver net polarization.^[12]^ When not counteracted, their efficiency, however, degrades when very strong magnetizations are manipulated in NMR probes with high‐quality‐factor resonators because of the interaction of the magnetization with their coils that induces the well known radiation damping.[^9^, ^12a^] Exactly this radiation damping can be used to turn the anti‐phase spin order of PHIP experiments into desired in‐phase magnetization via a RASER and we will show that this indeed happens spontaneously. A requirement for the RASER effect is negatively polarized spin order. Negatively polarized spins emit radio frequency energy which is absorbed by the NMR resonator and accumulated Q‐fold (quality factor of the coil) before dissipating. Normally, thermally polarized spins produce a small signal and require low noise amplifiers to be detected. Hyperpolarized spins, however, have four orders stronger magnitude, thus energy accumulated in the resonator is large enough to induce tangible electrical current and consequently a transverse magnetic field that acts back on the spins. This field has exactly the same frequency as the emissive lines in the spectrum and makes them irradiate even stronger that induces again the current in the resonator. Thus, emission of negatively polarized spins is stimulated until most of the spin energy is spent. Overall, the RASER is characterized by the radiation damping rate R_rd_, and sets in when *R*_rd_ is larger than the inverse transverse relaxation time:

(1) $$R{}_{\mathrm{rd}}>1/T{}_{2},$$

where

(2) $$R{}_{\mathrm{rd}}=1/2\mu {}_{0}\eta Q\gamma M{}_{0}$$

with *μ*_0_, *η*, *Q*, *γ*, *M*_0_ being the vacuum permeability, the filling factor of the coil, the quality factor of the detection circuit, the nuclear gyromagnetic ratio and the initial magnetization, respectively. PHIP produce both positive and negative magnetization but only negative magnetization emits radio frequency. The detected spectrum of transverse magnetization *M*_tr_ is depicted in Figure 1 C (RASER inset) and can be described by:^[9]^

(3) $$M_{tr}=M_{0}sech\left[R_{rd}\left(t-t_{0}\right)-ln\left(tan\frac{\theta_{0}}{2}\right)\right]\cos\omega t$$

with *ω* being the Larmor frequency and *θ*_0_ approaches 180°, corresponding to negative magnetization. The *θ*_0_ is disturbed from pure 180° because of noise fluctuations in the coil that slightly tilt the magnetization. This noise determines the time of the spontaneous onset of a RASER burst after negative magnetization is prepared (*t*_0_). This time is typically on the order of 100 ms, or alternatively, RASER can be triggered by a small flip angle pulse. The emission lasts for 1–100 ms depending on the initial magnetization: the larger the magnetization the shorter it is. At the end of the emission, thus, PHIP combined with RASER would deliver highly polarized proton medium with absorptive net magnetization suitable for SPINOE (Figure 1 D, SPINOE inset).

Here we propose the following experiment depicted in Figure 1 for which we suggest the acronym PRINOE (Para‐hydrogen and RASER Induced NOE). Highly polarized PHIP molecules at high field initiate a RASER leading to in‐phase magnetization. When brought into contact with other molecules in the liquid state a SPINOE will be observed. As a concrete experimental realization we react vinyl acetate‐d_6_ (VA) with pH_2_ to produce highly polarized proton spins in the resulting ethyl acetate‐d_6_ (EA). As previously predicted by us,^[12a]^ the concentration of the reactant has to be higher than 10 mM for this system to observe the spontaneous onset of a RASER‐induced signal. One such RASER burst in less than one second.^[12a]^ In order to synchronize with other events in a pulse sequence such as ^2^H‐decoupling or gradient switching, the RASER can be triggered by a radiofrequency pulse of small angle applied on protons. The *T*_2_ of the protons is long (on the order of seconds) and the linewidth is determined by the scalar splitting with deuterons which in turn can be removed by ^2^H decoupling. The relaxation time of the protons *T*_1_ is long enough (>90 s) to study slow processes like NOE. In addition, in the same work we demonstrated the way to control the onsets of radiation damping by applying a moderate gradient that allows precise kinetic investigation of the polarization transfer. Thus, PHIP on ethyl acetate‐d_6_ is a perfect model to study RASER induced effects caused by a hyperpolarized small molecule. As a target for polarization transfer solvent molecules with long proton relaxations are preferable to have enough time to accumulate substantial polarization. Solvent molecules are usually small and their liquid dynamics show a short correlation time. This is the so‐called extreme narrow limit and the sign of SPINOE polarization is expected to be negative compared to the source polarization. Note that in classical NOE the effect is called positive when radiofrequency saturation is applied and positive enhancement is observed.^[13]^ In the SPINOE case, however, high magnetization with the same sign as the Boltzmann magnetization will produce negative polarization on the target spins. The final polarization sign of the solvent molecules or generally molecules of interest can be changed by manipulating the polarization of the source spins by an inversion pulse. To prevent the onset of a RASER on highly polarized emissive EA, a gradient can be applied (see pulse sequence element D in Figure 1) which, however, should not influence the SPINOE process.

To compare the enhancement (*ϵ*) of Para‐hydrogen and RASER induced NOE the formula (*I*−*I*_0_)/*I*_th_ could be used, where *I*, *I*_0_, and *I*_th_ are signal integrals, respectively, for the SPINOE experiment with pH_2_, the same experiment but without adding pH_2_, and the thermally polarized spectrum. Subtracting *I*_0_ accounts for any relaxation or manipulation that can occur during the pulse sequence. After making the considerations above, we follow the described experimental Scheme in Figure 1 and investigate the effect on the solvent and on *N*‐acetyl tryptophane.

**Figure 2:**
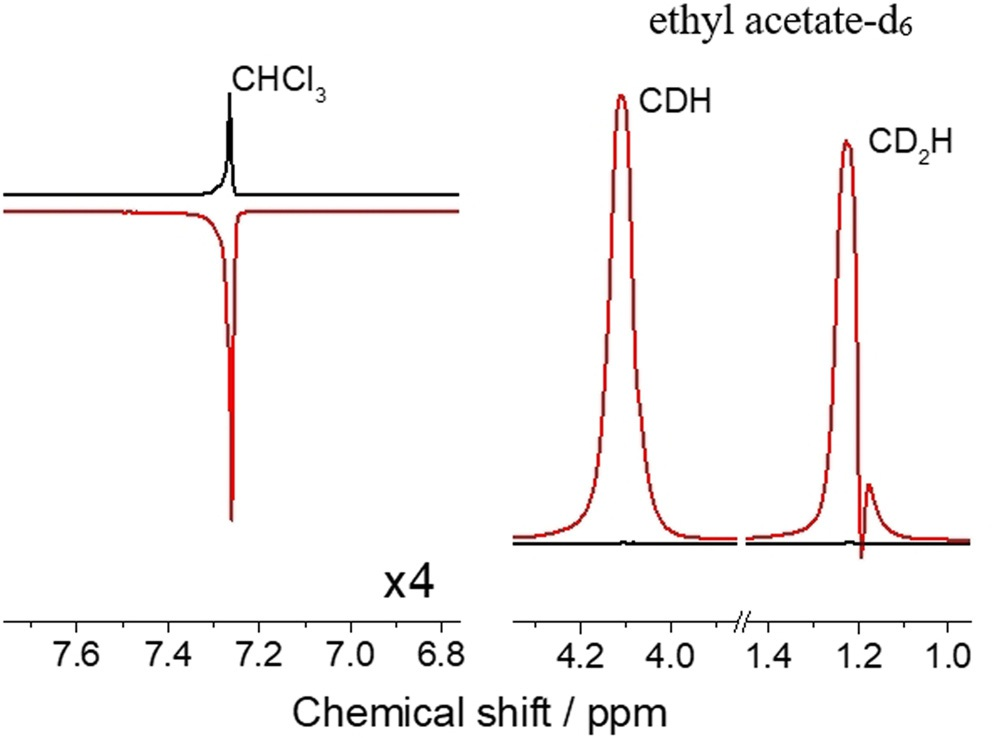
SPINOE on chloroform proton. PRINOE experiment following Figure 1 using a solution of 100 mM VA in 10 % protonated chloroform. PRINOE spectrum after 100 s of polarization transfer (red) in comparison to the thermally polarized spectrum (Black). Note the lines of highly polarized EA (right spectrum) broaden because of radiation damping but are in‐phase. The PRINOE enhancement on the chloroform proton is −4.4.

**Figure 3:**
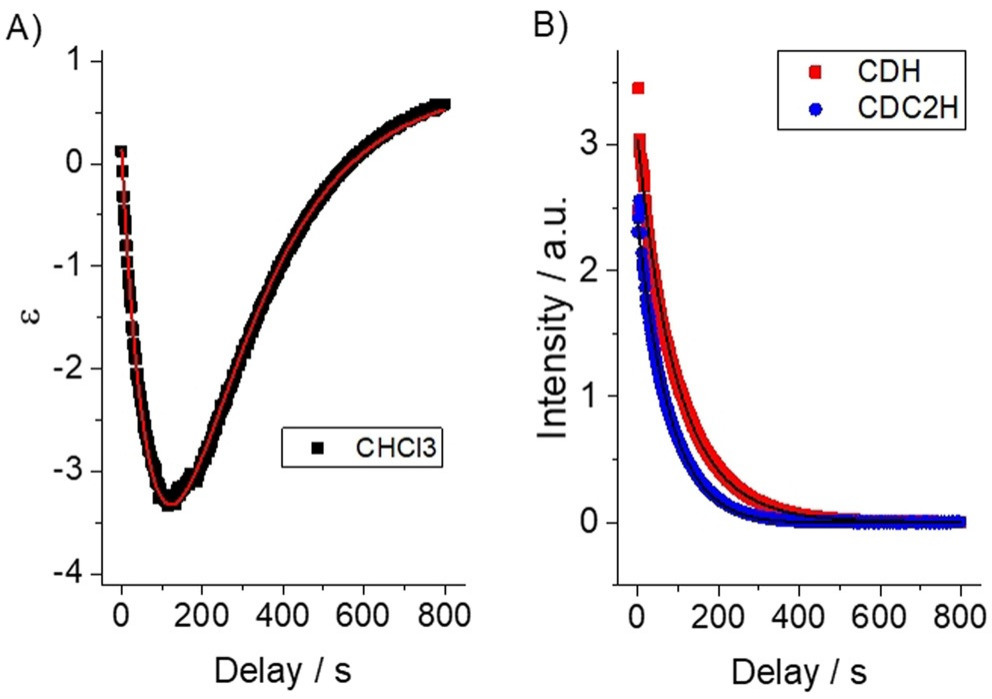
Kinetics of PRINOE. A) The solvent SPINOE enhancement *ϵ* and B) normalized EA intensity measured with 5° detection pulses and 2 s intervals. The decay times of EA are 97 and 75 s for CDH and CD_2_H protons, respectively. T_1_ of the thermally polarized CHCl_3_ on the same sample is 182±18 s. Filled circles indicate measured data and lines indicated simulated data, according to the numeric model described in section 5 of the SI.

For the experimental realization, we chose a solvent mixture of 10 % protonated and 90 % deuterated chloroform (CHCl_3_). In the chloroform molecule, a single proton can be found with a long longitudinal relaxation time (ca. 180 s). Para‐hydrogenation of 100 mM VA resulted in hyperpolarized EA that displays a RASER during one second and, thus, changes the PASADENA‐type anti‐phase polarization into pure net positive polarization. After 100 s of time evolution, a high absorptive signal of EA is observed together with the enhanced emissive signal of CHCl_3_ spins (Figure 2). SPINOE kinetics were measured on a single sample by applying a small flip angle pulse after variable delays following the RASER period (Figure 3). The negative enhancement of the solvent line increases and reaches a maximum peak intensity of −4.4 fold at ca. 100 s, corresponding to a polarization of *P*=−9.9×10^−5^. For analysis, the time‐traces for VA and CHCl_3_ shown in Figure 3 were subject to a simultaneous fitting according to a three‐spin model considering inter‐ and intramolecular dipolar cross‐relaxation. The SPINOE dynamics are well described by the relations describing transient NOE effects (as detailed in section 2 of the SI), which indicates that no further significant magnetization buildup occurs after the RASER period (panel C of Figure 1). The distance of minimum approach determining the rate for intermolecular cross‐relaxation in this case is *d*_kl_=(4.1±1.7) Å, when assuming a mutual self‐diffusion constants of *D*_kl_=2.9×10^−9^ m^2^ s^−1^. Further details of the fitting procedure are contained in section 5 of the SI.

The observed enhancement is relatively mild because of slow stochastic intermolecular cross relaxation in comparison to *T*_1_ relaxation. We anticipate, however, that the enhancement would grow almost proportionally to concentration of hyperpolarization‐source molecules and reaching theoretically another 100‐fold when pure para‐hydrogenated VA is used as a hybrid solvent and polarization source.

**Figure 4:**
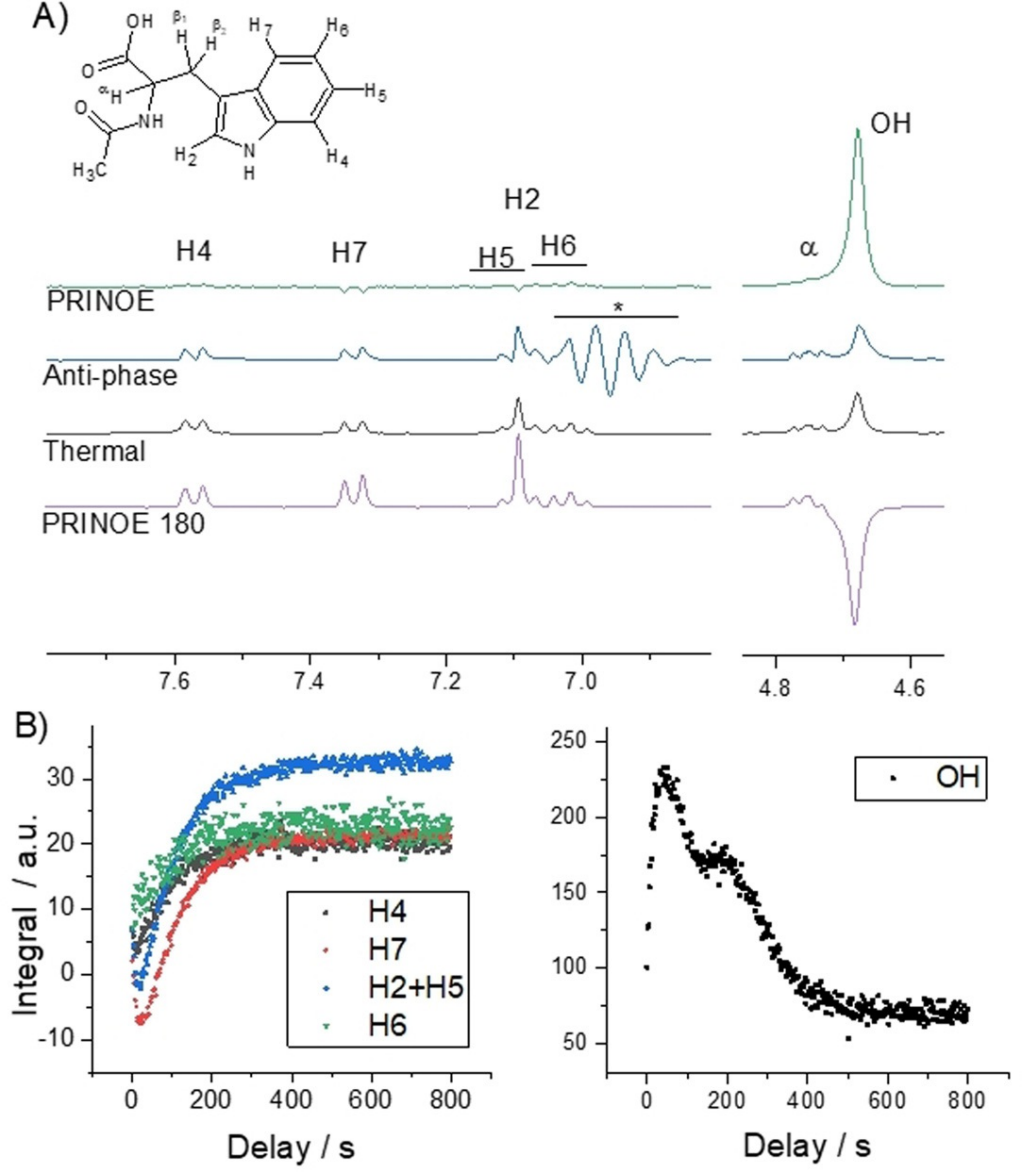
PRINOE on N‐acetyl‐L‐tryptophan. A) The spectra of PRINOE and PRINOE 180 in comparison to the thermally polarized spectrum and a spectrum without RASER but with the original PASADENA anti‐phase magnetization on EA. * denotes artifacts caused by overloading of the spectrometer receiver even with the smallest available receiver gain. B) Enhancement kinetics measured with 5° pulses and 2 s intervals after para‐hydrogenation, applying the RASER for 1 s and subsequently suppressing it with a gradient during another delay of 2 s. Most of the PRINOE kinetics of protons of Trp follow a bi‐exponential function with decay and rise time constants: 5 and 93 s for H4; 16 and 94 s for H7; 10 and 93 s for H2 with partly overlapping H5; 27 and 87 s for CH_3_ and a mono‐exponential rise with 97 s for H6. Exchangeable protons of the solution have complex kinetics with a 15 s rise time constant.

To demonstrate that the transfer of PHIP polarization is universal, several other solvents and solute combinations were polarized by PRINOE and results are collected in table S1 of the SI. Especially interesting is the case of *N*‐acetyl tryptophan (Trp) dissolved in [D_4_]MeOH (Figure 4) that had also been previously used in experiments studying hyperpolarization with a shuttle DNP device.^[14]^ We succeeded in enhancing the signal of the tryptophan derivative using PRINOE directly in solution. Trp has many different protons with various relaxation times, which makes it a good template to study the PRINOE phenomenon. The slowly relaxing spins have more time to accumulate signal enhancement via PRINOE, while the fast relaxing spins have marginal enhancements if any. The maximum signal boost is observed after ca. 25 s and all enhancements are summed in Table 1. Not only a negative SPINOE but also a positive effect is observed on exchangeable protons notably by OH‐protons of methanol. The positive enhancement is surprising as all molecules in solution are small and thus within the narrow NOE regime where a negative signal is expected. Particularly in this molecule the positive polarization cannot be explained by a relayed SPINOE transfer and chemical exchange. In each event the transfer occurs from highly polarized spins to less polarized ones. The protons of Trp are polarized less than OH‐protons and thus cannot cause it. To gain more insight we measured PRINOE in pure [D_4_]MeOH solvent under otherwise the same conditions. Positive SPINOE on OH‐proton (+4.1) as well as on the residual protons in the methyl group (+1.7) were observed. Explanations for the positive PRINOE can be the much slower tumbling rate or a different mechanism of NOE based on scalar relaxation: the positive intramolecular NOE was previously observed for in‐phase magnetization by Fukumi et al. on several alcohols^[15]^ and was also observed in other systems, exclusively intramolecularly.^[16]^

**Table 1:** Relaxation times, experimental enhancement factors (*ϵ*) and polarizations (*P*) of the tryptophan derivative and the solvent.^[a]^

		H4	H7	H2+H5	H6	OH	α, β1, β2	CD_2_HOD	CH_3_
T1/ s		4.4	8.5	7.0	3.6	115	<1.0	30.6	2.5
PRINOE	*ϵ*	−0.8	−1.4	−1.1	−0.6	2.9	0	0	−0.5
	*P*/ 10^−5^	+0.5	−0.8	−0.1	+0.7	+6.6	0	0	+1.0
PRINOE 180	*ϵ*	0.7	1.4	1.4	0.2	−2.8	0	−0.1	0.5
	*P*/ 10^−5^	+3.8	+5.4	+3.2	+2.7	−4.1	0	+2.0	+3.4

[a] The enhancements reported refer to *ϵ*=(*I*−*I*_0_)/*I*_th_, where *I* refers to the signal integral observed in the PRINOE experiment with pH_2_, *I*_0_ to the integral in the same experiment without adding pH_2_ and *I*_th_ to the integral in the thermally polarized spectrum.

We see the following possible ways to improve the PRINOE in the future. The challenge of masking signals of interest by the high polarization of EA can be suppressed during acquisition in the same way as it is done for water signals in protein studies. The stimulated emission occurs also after para‐hydrogenation of protonated precursors, which would be favorable compounds to use with respect to associated costs. The main hindrance, however, is the shorter relaxation times of the protonated molecules that shortens the time of high polarization and thus SPINOE. Only minor PRINOE was observed by using protonated VA. However, other molecules with longer relaxation would be useful here. For relatively low concentrations of hyperpolarized molecules a pulse sequence can be applied to distribute polarization after PHIP to only one proton. For example, the polarization was shifted completely to one proton of the ethyl group in EA in our previous work.^[12b]^ This can be advantageous in studying solvent‐solute interactions.

Liquid state hyperpolarization is important to study biological samples at native temperatures. Temperature‐Jump solution DNP,^[17]^ rapid‐melt DNP,^[18]^ dDNP,^[3f]^ Overhauser DNP^[19]^ and bullet‐DNP^[20]^ are a few examples aiming to achieve liquid state hyperpolarized samples for NMR. However, most of these techniques have difficulty bringing the hyperpolarization universally onto target molecules. The method we describe in the present work, the PRINOE, has the advantage as compared to most of the abovementioned methods, of always being in the liquid state without any phase transition from solid to liquid as is the case for most of them. Thus, its translation into a method of general applicability for studying biological samples clearly is of greatest interest and will be pursued in the future.

We would furthermore like to note that the RASER effect as used in the present work cannot be recycled: a single‐burst that creates positive net polarization. However, a stationary‐RASER as described by Appelt and co‐workers[^8^, ^21^] can deliver high polarization in a continuous manner and, thus, in return achieve a steady buildup of hyperpolarization on target molecules via SPINOE that we have described in the present work. This is currently suitable for SABRE at low field where the RASER was originally observed. This approach resembles the continuous solid‐state DNP driven hyperpolarization buildup and has a great potential as a practical method for liquid state hyperpolarized NMR. It would however require field cycling to detect samples at high field.

## Conclusion

Polarization enhancement of a broad range of target molecules was shown for the first time by combining PHIP, RASER and SPINOE methods forming the PRINOE. Although a 5° pulse was used in the experiments to precisely trigger the RASER event and with ^2^H decoupling applied, we also observed that the polarization transfer can occur completely without any external manipulation, hence spontaneously. Positive enhancement was obtained on exchangeable protons of the solution, methanol, while other protons of solutes show the negative signal in full accordance with being in the narrow limit of fast tumbling molecules. The enhancement sign can be inverted by applying a 180° pulse after the RASER and before the SPINOE step, dissipating the drawbacks of the OE's inherent reversal of hyperpolarization onto target molecules. This means that the signal enhancement is added to the thermally polarized sign rather than subtracted, ultimately leading to a higher gain in enhancement. In this way we have shown that signals can be enhanced in solvents and in an amino acid and we expect many more applications in the future that will help to solve structures of molecules with increased sensitivity.

## Conflict of interest

The authors declare no conflict of interest.

## Supporting information

As a service to our authors and readers, this journal provides supporting information supplied by the authors. Such materials are peer reviewed and may be re‐organized for online delivery, but are not copy‐edited or typeset. Technical support issues arising from supporting information (other than missing files) should be addressed to the authors.

Supporting Information
