# Supplementary material for: Spontaneous Enhancement of Magnetic Resonance Signals Using a RASER
Source: Angew Chem Int Ed Engl. 2021 Aug 13;60(38):20984–90. doi: 10.1002/anie.202108306 (PMC8518078; doi:10.1002/anie.202108306)
Supplement: Supplementary file 1 — Supporting Information [file ANIE-60-20984-s001.pdf]

Angewandte Chemie
Eine Zeitschrift der Gesellschaft Deutscher Chemiker

Supporting Information

Spontaneous Enhancement of Magnetic Resonance Signals Using a RASER

*Sergey Korchak, Lukas Kaltschnee, Riza Dervisoglu, Loren Andreas, Christian Griesinger, and Stefan Glöggler**

anie_202108306_sm_miscellaneous_information.pdf

1 Experimental Section

The experiments were performed using a Bruker 7 Tesla spectrometer Avance III HD equipped with a 5 mm probe with outer ^1H coil. The pH_2 with 99% content was delivered by a custom-made parahydrogen generator from ColdEdge Technologies, Inc.. The hyperpolarization transfer occurs in the liquid state at high field inside the cryomagnet in a SPINOE fashion. The experiments are depicted in Figures 1 of the main text as schemes and detailed in the following. Samples containing various solvents and optionally 100 mM N-acetyl tryptophan were investigated and are listed in Table S1. The source of polarization was a reaction of pH_2 with vinyl acetate-d_6 at 100 mM concentration in the presence of 4 mM of Rh catalyst ([1,4-Bis(diphenylphosphino)butane] (1,5-cyclooctadiene)rhodium(I) tetrafluoroborate), unless otherwise stated. All samples were deoxygenated before the experiments by replacing oxygen with N_2 gas. In the beginning of the experiment the samples were allowed to fully relax at 7 T. Then a gradient field causing 10 ppm of line broadening was applied and bubbling of pH_2 at 8 bar pressure was started. The hydrogenation reaction was allowed to occur for 20 s at 320 K temperature to ensure complete hydrogenation of vinyl acetate-d_6 into ethyl acetate-d_6. Afterwards the pH_2 pressure was released and nitrogen was bubbled for 2 s to stop further reaction and formation of bubbles in the sample. Subsequently, the gradient was switched off and ^2H radiofrequency decoupling applied. Together with an optional small B_1 pulse of 0.5 μs ^2H decoupling facilitates the stimulated emission of RASER[7b] which was allowed to happen for a total of 1 s. At this time point polarization transfer from positively polarized ethyl acetate-d_6 to the solvent and solute in liquid begins in a SPINOE manner. The samples stayed inside the cryomagnet and spectra showing the effect are detected after variable delay with a 5° or 90° radiofrequency pulse. Optionally the polarization of ethyl acetate-d_6 was inverted to negative with a 180° radiofrequency pulse after the RASER and before the SPINOE period. During the SPINOE period (D), a gradient field of 10 ppm was applied as well to prevent spontaneous emission, namely to suppress the RASER.

Table S1. Additionally tested solvents with enhancement values. 100mM of VA is used as precursor if not stated otherwise.

Composition	Enhancement
100% $CHCl_3$	-1.4
100% $CDCl_3$	~-3.7
100% Methanol-d4	OH +3.1, CHD_2 +0.7
100% $CHCl_3$ (protonated VA)	~-0.1
10% $CHCl_3$, 90% $CDCl_3$	-4.4
40% $CHCl_3$ 10% $CDCl_3$ 40% $C_2Cl_4H_2$ 10% $C_2Cl_4D_2$	Both solvents -2.5
10% $CHCl_3$ 40% $CDCl_3$ 10% $C_2Cl_4H_2$ 40% $C_2Cl_4D_2$	Both solvents -3.3
80% $C_2Cl_4H_2$ 20% $C_2Cl_4D_2$	-1.65
40% $CHCl_3$ 10% $CDCl_3$, 36% $C_2Cl_4H_2$, 9% $C_2Cl_4D_2$, 5% CD_3OD	~-1.3
5% CH_3OH 95% Acetone-d6	-0.4
Trp in 100% Acetone-d6	~-0.5, see also Table S2
Trp in 90% Acetone-d6, 10% $CHCl_3$	Trp ~-0.5, $CHCl_3$ =-5
Trp in 90% CD_3OD, 10% $CHCl_3$	$CHCl_3$=-0.76, OH=-0.34,<br>Trp~-0.5
Trp in 50% CD_3OD, 50% $CHCl_3$	$CHCl_3$=-1.2, OH=-0.4,<br>Trp~-0.5

Table S2. T_1-values and PRINOE enhancements measured in a sample containing 100 mM N-acetyl tryptophane in 100% acetone-d6.

	H4	H7	H2	H5 + H6	α	β1, β2	CH3	EA CDH	EA CD_2H	Acetone-d5
T1 / s	5.2	8.4	6.4	4.5	7.3	1.3	3.1	150	130	87
PRINOE	-0.5	-0.6	-0.4				-0.3			-2

2 Simulation & fitting of longitudinal cross-relaxation

2.1 Equation on motion

For simulation, we use the modified Bloch-equations for describing longitudinal cross-relaxation and chemical exchange in dynamic chemical equilibrium[1]

$$\frac{d\left(\boldsymbol{M}_z(t) - \boldsymbol{M}_{eq}\right)}{dt} = (\boldsymbol{K} - \boldsymbol{R}) \cdot \left(\boldsymbol{M}_z(t) - \boldsymbol{M}_{eq}\right) \tag{1}$$

which yields

$$\boldsymbol{M}_z(t) = e^{(\boldsymbol{K}-\boldsymbol{R})t}\left(\boldsymbol{M}_{z,0} - \boldsymbol{M}_{eq}\right) + \boldsymbol{M}_{eq}. \tag{2}$$

Hereby, $\boldsymbol{K}$ is a matrix of kinetic constants, $\boldsymbol{R}$ is the relaxation matrix and $\boldsymbol{M}_z(t)$, $\boldsymbol{M}_{z,0}$ and $\boldsymbol{M}_{eq}$ are vectors containing the system magnetizations at time point t, at time point zero and at thermal equilibrium, respectively.

The vector $\boldsymbol{M}_z(t)$ contains the magnetizations of all given groups k of chemically equivalent spins, which are given by

$$M_{z,k}(t) = N_k \gamma_k \hbar \sum_{m=-I_k}^{I_k} p_m\, m, \tag{3}$$

with N_k being the number of spins k per volume, γ_k the gyromagnetic ratio of spins k, $\hbar$ Planck's constant divided by 2π, I_k the spin quantum number of spins k, m the angular momentum quantum numbers of the $2I_k + 1$ eigenstates of the Zeeman operator, and p_m the populations of these respective eigenstates[2]. These populations can directly be related to the polarization P_k, which for spin-1/2 simply is $P_k = \frac{p_\alpha - p_\beta}{p_\alpha + p_\beta}$.

Normalizing to $p_\alpha + p_\beta = 1$, from which $\frac{(P_k+1)}{2} = p_\alpha$ and $\frac{(1-P_k)}{2} = p_\beta$ we find

$$M_{z,spin-1/2,k}(t) = \frac{N_k \gamma_k \hbar}{2}\left(\frac{(P_k + 1)}{2} - \frac{(1 - P_k)}{2}\right) = \frac{N_k \gamma_k \hbar}{2} P_k \tag{4}$$

For the following, we will decompose N_k as

$$N_k = c_k n_k N_a, \tag{5}$$

with c_k as the molar concentration of molecules carrying the nuclei k, n_k as the number of chemically equivalent nuclei k per molecule and N_a as Avogadro's constant.

The vector $\boldsymbol{M}_{eq}$ of equilibrium magnetizations, is obtained from Boltzmann statistics, with the high temperature approximation given being valid under the experimental conditions

$$M_{eq,k} = N_k \gamma_k \hbar \frac{\sum_m m \exp(m\gamma_k \hbar B_0 / k_B T)}{\sum_m \exp(m\gamma_k \hbar B_0 / k_B T)} \approx \frac{N_k \gamma_k^2 \hbar^2 I_k (I_k + 1) B_0}{3 k_B T}. \tag{6}$$

Here, B_0 is the magnetic flux density, k_B is the Boltzmann constant and T is the temperature.

2.2 Treatment of small flip-angle pulsing

During our experiments, the magnetizations $M_{z,k}(t)$ were probed by a series of small flip-angle pulses of $\phi = 5°$ in time intervals of 2 s. Since the scaling of the z-magnetization by these pulses is not negligible in our case, for each time point of the simulation, we scaled the magnetization of the previous point $\boldsymbol{M}_z(t - \Delta t)$ according to

$$\boldsymbol{M}_z{}'(t - \Delta t) = \cos\phi\, \boldsymbol{M}_z(t - \Delta t) \tag{7}$$

and then used the magnetization the equation of motion (*2*) to compute the current data point according to

$$\boldsymbol{M}_z(t) = \left(\boldsymbol{M}_z{}'(t - \Delta t) - \boldsymbol{M}_{eq}\right)e^{(\boldsymbol{K}-\boldsymbol{R})\Delta t} + \boldsymbol{M}_{eq}. \tag{8}$$

2.3 Treatment of chemical exchange

The matrix $\boldsymbol{K}$ contains the first-order rate constants of the chemical exchange happening in dynamic chemical equilibrium. For an exemplary reaction network involving the species A, B and C

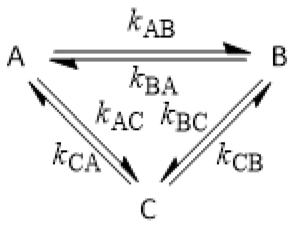

$\boldsymbol{K}$ is given by

$$\boldsymbol{K} = \begin{pmatrix} -(k_{AB} + k_{AC}) & +k_{BA} & +k_{CA} \\ +k_{AB} & -(k_{BA} + k_{BC}) & +k_{CB} \\ +k_{AC} & +k_{BC} & -(k_{CA} + k_{CB}) \end{pmatrix}, \tag{9}$$

and since we are in chemical equilibrium,

$$k_{ij} = k_{ji}\frac{c_{eq,j}}{c_{eq,i}}, \tag{10}$$

where $c_{eq,k}$ is the equilibrium concentration of species k.

2.4 Treatment of cross-relaxation

For our purpose, we explicitly include into $\boldsymbol{R}$ only terms describing contributions from dipolar couplings to relaxation. We include the terms for intramolecular relaxation under isotropic molecular tumbling, and for intermolecular relaxation under isotropic diffusion, neglecting possible effects of transient complex formation in solution.

To this end, we express $\boldsymbol{R}$ as the sum of the individual relaxation terms

$$\boldsymbol{R} = \boldsymbol{R}_{intra} + \boldsymbol{R}_{inter} + \boldsymbol{R}_{leakage}, \tag{11}$$

were $\boldsymbol{R}_{intra}$ contains the rates of relaxation due to the variation of intramolecular dipolar couplings by molecular reorientation, $\boldsymbol{R}_{inter}$ contains the rates of relaxation caused by variations of the intermolecular dipolar couplings by molecular diffusion and $\boldsymbol{R}_{leakage}$ is a diagonal matrix containing phenomenological leakage rates ρ_k^*, which should take into account all spin-lattice relaxation due to all relaxation mechanisms not explicitly considered here.

2.4.1 Intramolecular relaxation due to dipolar couplings

For constructing the full matrix $\boldsymbol{R}_{intra}$, the contributions from all intramolecular dipole-dipole interactions are collected. Pairwise interactions can be between nuclei belonging to the same group k of chemically equivalent spins (case of like spins) or between nuclei belonging to two different groups k and l (case of unlike spins). We hereby follow a notation similar to that used in ref. [3], with

$$J_{ij}(\omega) = \frac{\tau_{c,ij}}{1 + \left(\omega\tau_{c,ij}\right)^2}, \tag{12}$$

where $\tau_{c,kl}$ is the correlations time modulating the i and j interaction and

$$q_{ij} = \frac{1}{10}\gamma_i^2\gamma_j^2\hbar^2\langle r_{ij}^{-6}\rangle\left(\frac{\mu_0}{4\pi}\right)^2 \tag{13}$$

where $\langle r_{ij}^{-6}\rangle$ is the time-average over the inverse sixth power of the internuclear distance (assuming faster molecular tumbling than conformational interconversion) and where μ_0 is the vacuum permeability.

Interactions between nuclei belonging to the same group k of chemically equivalent spins only contribute to the element R_{kk} with

$$R_{kk,intra,like} = 2(n_k - 1)\left(W_1^{kk} + W_2^{kk}\right) \tag{14}$$

whereas interactions between nuclei of different groups k and l contribute to the elements R_{kk}, R_{ll}, R_{kl} and R_{lk}, according to

$$R_{kk,intra,unlike} = n_l\left(W_0^{kl} + 2W_1^{kl} + W_2^{kl}\right) \tag{15}$$

$$R_{ll,intra,unlike} = n_k\left(W_0^{lk} + 2W_1^{lk} + W_2^{lk}\right) \tag{16}$$

$$R_{kl,intra,unlike} = n_k\left(W_2^{kl} - W_0^{kl}\right) \tag{17}$$

$$R_{lk,intra,unlike} = n_l\left(W_2^{lk} - W_0^{lk}\right). \tag{18}$$

The transition probabilities are hereby given by[2,3]

$$W_1^{ij} = 2I_j\left(I_j + 1\right)q_{ij}J_{ij}(\omega_i) \tag{19}$$

$$W_0^{ij} = \frac{4}{3}I_i(I_i + 1)q_{ij}J_{ij}\left(\omega_i - \omega_j\right) \tag{20}$$

$$W_2^{ij} = 8I_i(I_i + 1)q_{ij}J_{ij}\left(\omega_i + \omega_j\right). \tag{21}$$

2.4.2 Intermolecular relaxation due to dipolar couplings

The rate of intermolecular relaxation is modulated the mutual (translational) self-diffusion constants D_{ij} of the two molecules involved and by the so-called distance of closest approach d_{ij} of the two nuclei. We assume, that the mutual self-diffusion constants D_{ij} can be expressed by the self-diffusion coefficients D_i and D_j of the individual molecules according to

$$D_{ij} = \tfrac{1}{2}\left(D_i + D_j\right). \tag{22}$$

The distance of closest approach d_{ij} is somehow vaguely defined, but a lower bound estimate can be obtained from the van der Waals radii of the atoms involved.

For constructing the matrix $\boldsymbol{R}_{inter}$ describing intermolecular relaxation due to diffusion, contributions from like spins and contributions from unlike spins need to be considered, as previously described for the intramolecular case.

In the limiting case, where $\omega d_{ij}^2 \ll 2D_{ij}$, which usually is realized for low-viscosity solvents, the interaction between like spins once again only contributes to R_{kk}, and is given by[2]

$$R_{kk,inter,like} = \frac{8\pi}{15} I_k(I_k+1)\gamma_k^4\hbar^2 \frac{N_k}{d_{kk}D_{kk}}\left(\frac{\mu_0}{4\pi}\right)^2. \tag{23}$$

For unlike spins, the contributions are

$$R_{kk,inter,unlike} = \frac{16\pi}{45} I_l(I_l+1)\gamma_k^2\gamma_l^2\hbar^2 \frac{N_l}{d_{kl}D_{kl}}\left(\frac{\mu_0}{4\pi}\right)^2 \tag{24}$$

$$R_{ll,inter,unlike} = \frac{16\pi}{45} I_k(I_k+1)\gamma_k^2\gamma_l^2\hbar^2 \frac{N_k}{d_{kl}D_{kl}}\left(\frac{\mu_0}{4\pi}\right)^2 \tag{25}$$

$$R_{kl,inter,unlike} = \frac{8\pi}{45} I_k(I_k+1)\gamma_k^2\gamma_l^2\hbar^2 \frac{N_k}{d_{kl}D_{kl}}\left(\frac{\mu_0}{4\pi}\right)^2 \tag{26}$$

$$R_{lk,inter,unlike} = \frac{8\pi}{45} I_l(I_l+1)\gamma_k^2\gamma_l^2\hbar^2 \frac{N_l}{d_{kl}D_{kl}}\left(\frac{\mu_0}{4\pi}\right)^2. \tag{27}$$

3 SPINOE for a two-spin system

Descriptions of cross-relaxation within two-spin systems are widespread amongst the literature. Particularly illustrative descriptions are given in chapter 5.1 of ref. [4] and in ref. [3].

3.1 SPINOE driven by translational diffusion in an ideal two-spin system

The Solomon equations[5] for describing longitudinal relaxation of the longitudinal magnetizations I_z and S_z of a two-spin system, as the explicit form of equation (*2*) for two spins, are given by

$$\begin{aligned}\frac{dI_z}{dt} &= -\rho_I\left(I_z - I_{eq}\right) - \sigma_{IS}\left(S_z - S_{eq}\right)\\ \frac{dS_z}{dt} &= -\sigma_{SI}\left(I_z - I_{eq}\right) - \rho_S\left(S_z - S_{eq}\right),\end{aligned} \qquad (28)$$

with the identifications

$$\boldsymbol{M}_z(t) = \begin{pmatrix} I_z(t) \\ S_z(t) \end{pmatrix}, \boldsymbol{M}_{eq} = \begin{pmatrix} I_{eq} \\ S_{eq} \end{pmatrix} \text{ and } (\boldsymbol{K}-\boldsymbol{R}) = -\begin{pmatrix} \rho_I & \sigma_{IS} \\ \sigma_{SI} & \rho_s \end{pmatrix}. \qquad (29)$$

We will use S as the hyperpolarized source of non-thermal magnetization and I as the spin we are aiming to hyperpolarize.

Considering only intermolecular relaxation and no chemical exchange ($\boldsymbol{K} = \boldsymbol{0}$), we find

$$\begin{aligned}\boldsymbol{R} &= \begin{pmatrix} \rho_I & \sigma_{IS} \\ \sigma_{SI} & \rho_S \end{pmatrix}\\ &= \begin{pmatrix} R_{II,inter,like} + R_{II,inter,unlike} + \rho_I^* & R_{IS,inter,unlike} \\ R_{SI,inter,unlike} & R_{SS,inter,like} + R_{SS,inter,unlike} + \rho_S^* \end{pmatrix},\end{aligned} \qquad (30)$$

where ρ_I^* and ρ_S^* are the rates of undefined leakage for I and S respectively.

In the low viscosity limit ($\omega d_{ij}^2 \ll 2D_{ij}$),

$$\sigma_{IS} = \frac{I_I(I_I+1)}{I_S(I_S+1)}\frac{N_I}{N_S}\sigma_{SI}. \qquad (31)$$

(To avoid confusion, note at this point that the reverse relationship is obtained, when in equation (*28*) we are taking I_z and S_z as the z-components of the $\boldsymbol{I}$ and $\boldsymbol{S}$ spins as done in ref. [6], instead of the taking I_z and S_z as the z-magnetizations of the two spin-groups.)

For evaluating $e^{(\boldsymbol{K}-\boldsymbol{R})t}$ in equation (*3*), we use

$$e^{(\boldsymbol{K}-\boldsymbol{R})t} = \boldsymbol{V}\begin{pmatrix} e^{\lambda_1 t} & 0 \\ 0 & e^{\lambda_2 t} \end{pmatrix}\boldsymbol{V}^{-1}, \qquad (32)$$

where λ_1 and λ_2 are the eigenvalues of $(\boldsymbol{K}-\boldsymbol{R})$ and $\boldsymbol{V}$ contains the corresponding eigenvectors. As solutions, we find

$$\lambda_1 = -\frac{1}{2}\left[\rho_I + \rho_S + \sqrt{(\rho_I - \rho_S)^2 + 4\sigma_{IS}\sigma_{SI}}\right] \qquad (33)$$

$$\lambda_2 = -\frac{1}{2}\left[\rho_I + \rho_S - \sqrt{(\rho_I - \rho_S)^2 + 4\sigma_{IS}\sigma_{SI}}\right] \quad (34)$$

$$\boldsymbol{V} = \begin{pmatrix} -\sigma_{IS} & \lambda_2 + \rho_S \\ \lambda_1 + \rho_I & -\sigma_{SI} \end{pmatrix} \quad (35)$$

$$\boldsymbol{V^{-1}} = \frac{1}{\sigma_{IS}\sigma_{SI} - (\lambda_1 + \rho_I)(\lambda_2 + \rho_S)} \begin{pmatrix} -\sigma_{SI} & -\lambda_2 - \rho_S \\ -\lambda_1 - \rho_I & -\sigma_{IS} \end{pmatrix}, \quad (36)$$

which can be substituted into (*3*), to get

$$\begin{pmatrix} I_z(t) \\ S_z(t) \end{pmatrix} = \boldsymbol{V} \begin{pmatrix} e^{\lambda_1 t} & 0 \\ 0 & e^{\lambda_2 t} \end{pmatrix} \boldsymbol{V^{-1}} \left(\begin{pmatrix} I_{z,0} \\ S_{z,0} \end{pmatrix} - \begin{pmatrix} I_{eq} \\ S_{eq} \end{pmatrix} \right) + \begin{pmatrix} I_{eq} \\ S_{eq} \end{pmatrix}. \quad (37)$$

For our purpose, we can assume, that $\frac{4\sigma_{IS}\sigma_{SI}}{(\rho_I - \rho_S)^2} \ll 1$, a condition where we obtain

$$\lambda_1 \approx -\rho_I - \frac{\sigma_{IS}\sigma_{SI}}{(\rho_I - \rho_S)} \quad (38)$$

$$\lambda_2 \approx -\rho_S + \frac{\sigma_{IS}\sigma_{SI}}{(\rho_I - \rho_S)} \quad (39)$$

Further assuming, that $I_{z,0} = I_{eq}$ we obtain

$$\begin{aligned} &I_z(t) \\ &\approx I_{eq} + \left(S_{z,0} - S_{eq}\right) \frac{\sigma_{IS}}{\rho_I - \rho_S} \frac{1}{1 + \frac{\sigma_{IS}\sigma_{SI}}{(\rho_I - \rho_S)^2}} \left[\exp\left(\left(-\rho_I - \frac{\sigma_{IS}\sigma_{SI}}{(\rho_I - \rho_S)} \right) t \right) \right. \\ &\qquad \left. - \exp\left(\left(-\rho_S + \frac{\sigma_{IS}\sigma_{SI}}{(\rho_I - \rho_S)} \right) t \right) \right] \\ &\approx I_{eq} + \left(S_{z,0} - S_{eq}\right) \frac{\sigma_{IS}}{\rho_I - \rho_S} \left[\exp\left(\left(-\rho_I - \frac{\sigma_{IS}\sigma_{SI}}{(\rho_I - \rho_S)} \right) t \right) - \exp\left(\left(-\rho_S + \frac{\sigma_{IS}\sigma_{SI}}{(\rho_I - \rho_S)} \right) t \right) \right] \end{aligned} \quad (40)$$

and

$$\begin{aligned} &S_z(t) \\ &\approx S_{eq} + \left(S_{z,0} - S_{eq}\right) \left[\frac{1}{1 + \frac{\sigma_{IS}\sigma_{SI}}{(\rho_I - \rho_S)^2}} \exp\left(\left(-\rho_S + \frac{\sigma_{IS}\sigma_{SI}}{(\rho_I - \rho_S)} \right) t \right) \right. \\ &\qquad \left. + \frac{1}{1 + \frac{(\rho_I - \rho_S)^2}{\sigma_{IS}\sigma_{SI}}} \exp\left(\left(-\rho_I - \frac{\sigma_{IS}\sigma_{SI}}{(\rho_I - \rho_S)} \right) t \right) \right] \\ &\approx S_{eq} + \left(S_{z,0} - S_{eq}\right) \exp\left(\left(-\rho_S + \frac{\sigma_{IS}\sigma_{SI}}{(\rho_I - \rho_S)} \right) t \right). \end{aligned} \quad (41)$$

The enhancement on I, as defined by $\left(I_z - I_{eq}\right)/I_{eq}$ is obtained as

$$\begin{aligned} &\frac{\left(I_z(t) - I_{eq}\right)}{I_{eq}} = \frac{\left(I_z(t) - I_{eq}\right)}{S_{eq}} \frac{N_S \gamma_S^2}{N_I \gamma_I^2} \frac{I_S(I_S + 1)}{I_I(I_I + 1)} \\ &\approx \frac{\left(S_{z,0} - S_{eq}\right)}{S_{eq}} \frac{N_S \gamma_S^2}{N_I \gamma_I^2} \frac{I_S(I_S + 1)}{I_I(I_I + 1)} \frac{\sigma_{IS}}{\rho_I - \rho_S} \\ &* \left[\exp\left(\left(-\rho_I - \frac{\sigma_{IS}\sigma_{SI}}{(\rho_I - \rho_S)} \right) t \right) - \exp\left(\left(-\rho_S + \frac{\sigma_{IS}\sigma_{SI}}{(\rho_I - \rho_S)} \right) t \right) \right] \end{aligned} \quad (42)$$

Further simplifying, under the restrictions, that $I_I = I_S$, $\gamma_I = \gamma_S$, $\frac{\sigma_{IS}\sigma_{SI}}{(\rho_I-\rho_S)} \ll \rho_I$ and $\frac{\sigma_{IS}\sigma_{SI}}{(\rho_I-\rho_S)} \ll \rho_S$ one finds the much simpler form

$$\frac{\left(I_z(t) - I_{eq}\right)}{I_{eq}} \approx \frac{\left(S_{z,0} - S_{eq}\right)}{S_{eq}} \frac{N_S}{N_I} \frac{\sigma_{IS}}{\rho_I - \rho_S} [\exp(-\rho_I t) - \exp(-\rho_S t)], \quad (43)$$

where $\left(S_{z,0} - S_{eq}\right)/S_{eq} = \epsilon_s$ is the enhancement factor from hyperpolarization, achieved for spin *S*.

From (*37*) we find that when setting $I_{z,0} = I_{eq}$ the time for the maximum enhancement is

$$t_{max} = \frac{1}{\lambda_1 - \lambda_2} \ln\left(\frac{\lambda_2}{\lambda_1}\right) \approx -\frac{1}{(\rho_I - \rho_S)} \ln\left(\frac{\rho_S}{\rho_I}\right). \quad (44)$$

4 Size estimate of the SPINOE between protons

In the Solomon equations for the evolution of longitudinal magnetization (equation 28), we will describe the cross-relaxation rates σ_{kl} by the terms for driven by translational diffusion (equations (*26*) and (*27*)), as described in equation (*30*). This description is valid in the extreme narrowing limit for translational diffusion, which is the limit usually valid in low viscosity solutions, if at least one of the compounds is a small molecule (for further details, see chapter 2.4.2 of the SI).

$$\sigma_{IS} = R_{kl,inter,unlike} = \frac{8\pi}{45} I_k(I_k + 1)\gamma_k^2 \gamma_l^2 \hbar^2 \frac{N_k}{d_{kl} D_{kl}} \left(\frac{\mu_0}{4\pi}\right)^2 \quad (26)$$

For a size-estimate of this cross-relaxation rate for a pair of ^{1}H-nuclei, let us start off with the system of hyperpolarized ethyl acetate-d6 in chloroform, discussed in Fig. 2 of the main article.

We assume that it is appropriate to combine the magnetizations of both ^{1}H on the ethyl acetate-d6 into S_z, which should be a reasonably good approximation as long as the two protons do not feature significantly different intermolecular cross-relaxation rates to the CHCl3. For comparability with Fig. 2 we will therefore use $N_S = 0.2\ M$ and $N_I = 0.1\ M$ for $CHCl_3$.

The distance of minimal approach is hard to define, but let's assume that a lower bound for $d_{kl}/2$ is given by the van der Waals radius of hydrogen (~ 1.2 Å).

We scaled the self-diffusion coefficients of chloroform at 25°C ($2.6*10^{-9}$ m^2 s^{-1})[7] and diffusion coefficient of ethyl acetate in chloroform at infinite dilution ($2.02*10^{-9}$ m^2 s^{-1})[8] by the decrease in viscosity for chloroform from 25°C to 50°C ($\eta_{25°C}(CHCl_3) = 0.537\ mPa\ s, \eta_{50°C}(CHCl_3) = 0.427\ mPa\ s$)[8] and by the temperature according to the Stokes-Einstein relation, to estimate the mutual diffusion coefficient $D_{kl} \approx 2.9 * 10^{-9} m^2/s$.

With these values, we find $\sigma_{kl} \approx c_k * 2 * 10^{-4}\ l\ mol^{-1} s^{-1}$, where c_k is the molar concentration of k. From the smallness of this value, it can be seen, that for free diffusion of small molecules in low viscosity solutions, the SPINOE mediated by translational diffusion will be very weak. Even for concentrations in the molar range, the corresponding cross-relaxations σ_{IS} and σ_{SI} will usually be much smaller than the auto-relaxation rates $\rho_k \rho_I$ and $\rho_S \rho_l$ (as estimated from typical T_1 for ^{1}H), and thus the expected transfer efficiencies will be low for small molecules in low-viscosity solutions.

Taking the example of a hyperpolarized spin *S* with $\rho_S \approx (85s)^{-1}$ as a value close to the average ^{1}H-T_1 for the protons in the ethyl acetate-d6 used later as hyperpolarization source and assuming for spin *I* of the ^{1}H-T_1 of $CHCl_3$ measured ($\rho_I \approx (180s)^{-1}$), we find the timepoint of maximum enhancement to be $t_{max} \approx 120s$ (see equation(*44*)). With this value we estimate that the maximum enhancement for $CHCl_3$ that could be achieved with a theoretical 100% polarization on *S* at $t = 0$ for this system is around $\varepsilon_{CHCl3,max} \approx -77$ (using equation S42), corresponding to a polarization of $P = 1.7*10^{-3}$. Numerical simulations for a three-spin-1/2 system also considering inter- and intramolecular cross-relaxation for ethyl acetate yields $\varepsilon_{CHCl3,max} \approx -73$ ($P = 1.6*10^{-3}$), which is in reasonable agreement, given the drastic simplification introduced in the two-spin-1/2 treatment. Thus, for target compounds with very long ^{1}H-T_1, such as chloroform, also notable enhancements can in principle be achieved.

When assuming $\rho_I \approx (5s)^{-1}$ as a more typical ^{1}H-T_1 for a small organic molecule such as the N-acetyl-tryptophan also discussed, we find the timepoint of maximum enhancement to be $t_{max} \approx 15s$ (equation S43). Using $D_{kl} \approx 2 * 10^{-9} m^2/s$, which probably is a more realistic estimate for the system discussed in Figure 4 of the main article, and again using $N_S = 0.2M$ and $N_I = 0.1M$, the maximum enhancement we would expect for a system with such shorter ^{1}H-T_1 to be around $\varepsilon_{I,max} \approx -15$ ($P = 3.4*10^{-4}$), when starting from 100% polarization on *S*.

Despite the relatively inefficient polarization transfer in low viscosity solvents through intermolecular NOE by translational diffusion, notable enhancements can be achieved with para-hydrogen enhanced substrates, due to the very high proton polarizations, that can be achieved these days.

5 Data Fitting

Fitting of the data shown in Figure 2 of the main article to equation (*2*) was performed using a custom script in Matlab® R2020b ((9.9.0.1467703) © 1984-2020 The MathWorks, Inc.).

We only analyzed the transfer of magnetization and the magnetization decay after conversion of the initial spin order into magnetization via the RASER. As a simplification, we assume that at the start of data acquisition, the PHIP reaction as well as the RASER have completely ceased and that only pure z-magnetization remains.

For all points of the experimental data, the simulated data was computed from the previous data point using equation (*8*), explicitly taking into account the magnetization scaling by the 5°-pulsing used.

Least squares fits were performed for three different datasets with initial concentration of vinyl acetate-d_6 of 10mM, 100mM and 200mM.

For the fits shown in Figure 2, simulations were performed for a system of three groups of spin-1/2, namely the H of $CHCl_3$, the methylene-H of ethyl acetate-d_6 and the methyl-H of ethyl acetate-d_6. Contributions from all other spins were condensed into the leakage rates ρ_k^*.

For each dataset, the distance of minimum approach was fitted as a global parameter condensing all possible d_{kl} within the system, to avoid linear dependencies.

Table S3. Summary of fixed and varied parameters during fitting.

Dataset	parameter	H of CHCl3	methylene-H of ethyl acetate-d6	methyl-H of ethyl acetate-d6
fixed parameters				
c_0(VA) = 10 mM		1.24	0.01	
c_0(VA) = 100 mM	c_k [M]	1.24	0.1	
c_0(VA) = 200 mM		1.24	0.15[a]	
	τ_C [ps]	1.6	2.0	
all datasets	D_k [10^{-9} m² s^{-1}]	3.5	2.3	
	$\langle r_{ij}^{-6} \rangle$ [Å]	-	2.624[b]	
fitted parameters				
c_0(VA) = 10 mM	ρ_k^* [10^{-3} s^{-1}]	2.7 ± 0.9	3.32 ± 0.16	4.73 ± 0.18
	d_{kl} [Å]	3.6 ± 1.4		
c_0(VA) = 100 mM	ρ_k^* [10^{-3} s^{-1}]	2.3 ± 1.3	3.12 ± 0.14	4.45 ± 0.17
	d_{kl} [Å]	4.1 ± 1.7		
c_0(VA) = 200 mM	ρ_k^* [10^{-3} s^{-1}]	2.7 ± 1.3	3.00 ± 0.13	4.40 ± 0.15
	d_{kl} [Å]	3.8 ± 1.4		

[a]: Product did not react to completeness. Used concentration measured after SPINOE experiment. [b]: Estimated from a structure optimized at B3LYP/6-31G(d,p) level, using Gaussian® 09 (rev C.01).[8]

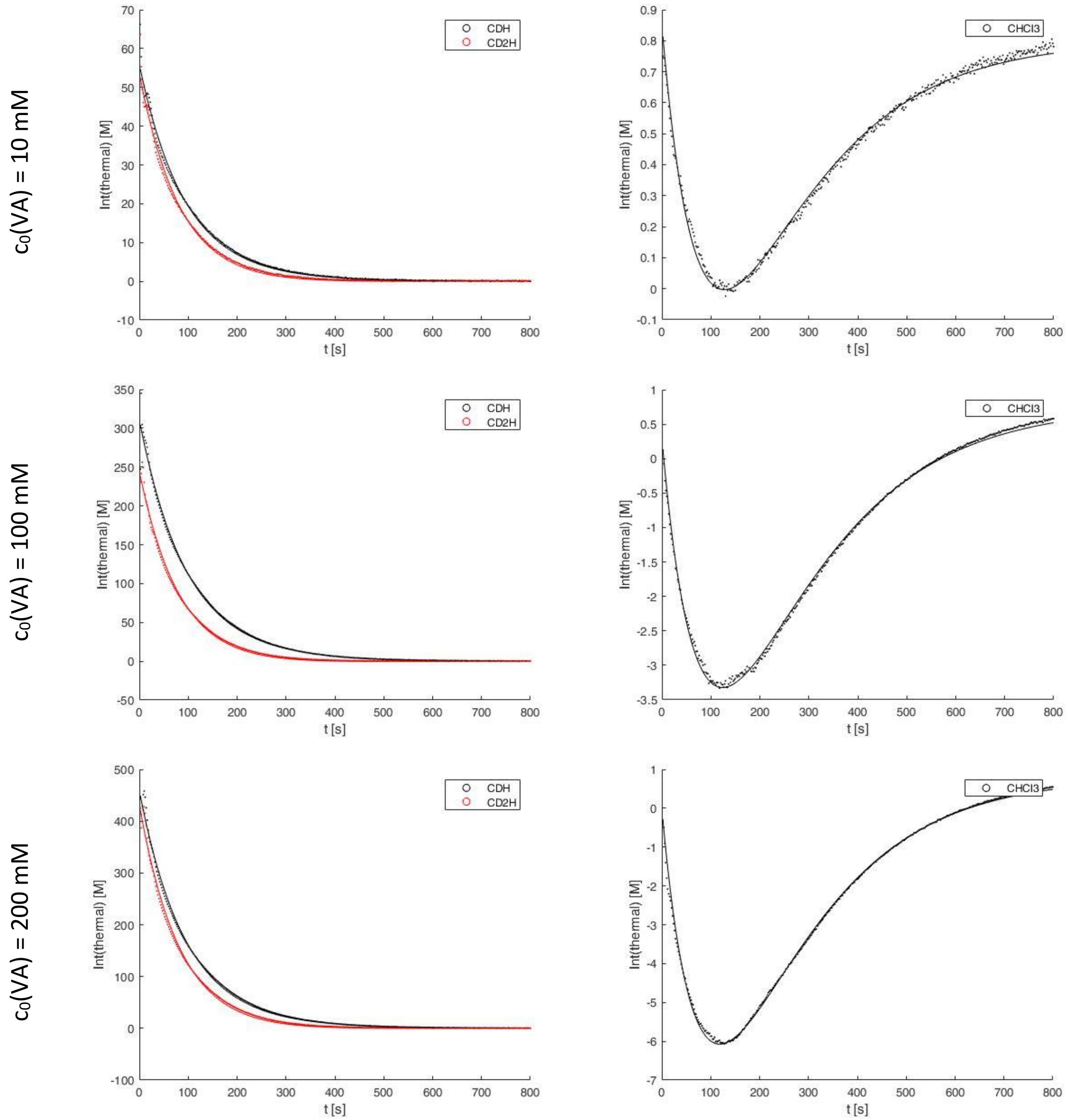

Figure S1. SPINOE kinetics measured for $CHCl_3$ for different initial concentrations of vinyl acetate-d_6 (VA). The vertical axis shows signal integrals, normalized to the expected thermal signal for a 1M compound (i.e.: $c_k \frac{P_k}{P_{eq}}$).

6 References

1. G. B. Richard R. Ernst, Alexander Wokaun, *Principles of Nuclear Magnetic Resonance in One and Two Dimensions*, Clarendon Press, Oxford, 1987.
2. A. Abragam, *The Principles of Nuclear Magnetism*, Oxford University Press, Oxford, 1961.
3. S. Macura and R. R. Ernst, *Molecular Physics*, 1980, **41**, 95-117.
4. J. Cavanagh, N. J. Skelton, W. J. Fairbrother, M. Rance and A. G. Palmer III, *Protein NMR Spectroscopy: Principles and Practice*, Academic Press, Amsterdam, 2 edn., 2006.
5. I. Solomon, *Physical Review*, 1955, **99**, 559-565.
6. G. Navon, Y.-Q. Song, T. Rõõm, S. Appelt, R. E. Taylor and A. Pines, *Science*, 1996, **271**, 1848-1851.
7. V. A. Golubev, D. L. Gurina and R. S. Kumeev, *Russian Journal of Physical Chemistry A*, 2018, **92**, 75-78.
8. *CRC Handbook of Chemistry and Physics*, CRC Press/Taylor & Francis, Boca Raton, FL, 101st Edition (Internet Version 2020) edn., 2020.
9. Gaussian 09, Revision C.01,
M. J. Frisch, G. W. Trucks, H. B. Schlegel, G. E. Scuseria, M. A. Robb, J. R. Cheeseman, G. Scalmani, V. Barone, B. Mennucci, G. A. Petersson, H. Nakatsuji, M. Caricato, X. Li, H. P. Hratchian, A. F. Izmaylov, J. Bloino, G. Zheng, J. L. Sonnenberg, M. Hada, M. Ehara, K. Toyota, R. Fukuda, J. Hasegawa, M. Ishida, T. Nakajima, Y. Honda, O. Kitao, H. Nakai, T. Vreven, J. A. Montgomery, Jr., J. E. Peralta, F. Ogliaro, M. Bearpark, J. J. Heyd, E. Brothers, K. N. Kudin, V. N. Staroverov, T. Keith, R. Kobayashi, J. Normand, K. Raghavachari, A. Rendell, J. C. Burant, S. S. Iyengar, J. Tomasi, M. Cossi, N. Rega, J. M. Millam, M. Klene, J. E. Knox, J. B. Cross, V. Bakken, C. Adamo, J. Jaramillo, R. Gomperts, R. E. Stratmann, O. Yazyev, A. J. Austin, R. Cammi, C. Pomelli, J. W. Ochterski, R. L. Martin, K. Morokuma, V. G. Zakrzewski, G. A. Voth, P. Salvador, J. J. Dannenberg, S. Dapprich, A. D. Daniels, O. Farkas, J. B. Foresman, J. V. Ortiz, J. Cioslowski, and D. J. Fox, Gaussian, Inc., Wallingford CT, 2010.
